# Overcoming the Challenges of Water, Waste and Climate Change in Asian Cities

**DOI:** 10.1007/s00267-019-01137-y

**Published:** 2019-02-22

**Authors:** Annisa Noyara Rahmasary, Suzanne Robert, I-Shin Chang, Wu Jing, Jeryang Park, Bettina Bluemling, Stef Koop, Kees van Leeuwen

**Affiliations:** 1 Copernicus Institute of Sustainable Development and Innovation, Utrecht University, Utrecht, The Netherlands; 2 Water Systems and Global Change Group, Wageningen University & Research, Wageningen, The Netherlands; 3 School of Ecology and Environment, Inner Mongolia University, Hohhot, China; 4 College of Environmental Science and Engineering, Nankai University, Tianjin, China; 5 School of Urban and Civil Engineering, Hongik University, Seoul, Republic of Korea; 6 KWR Watercycle Research Institute, Nieuwegein, The Netherlands

**Keywords:** Water governance, Water management, Climate change, Cities, Solid waste, Wastewater, SDG6

## Abstract

Unprecedented challenges in urban management of water, waste and climate change—amplified by urbanisation and economic growth—are growing in Asia. In this circumstance, cities need to be aware of threats and opportunities to improve their capacity in addressing these challenges. This paper identifies priorities, barriers and enablers of these capacities. Through the City Blueprint^®^ Approach—an integrated baseline assessment of the urban water cycle—11 Asian cities are assessed. Three cities are selected for an in-depth governance capacity analysis of their challenges with a focus on floods. Solid waste collection and treatment and access to improved drinking water and sanitation can be considered priorities, especially in cities with considerable slum populations. These people are also disproportionately affected by the impacts of climate-related hazards. The high variation of water management performance among Asian cities shows high potential for city-to-city learning by sharing best practices in water technology and governance. Combining interventions, i.e., by exploring co-benefits with other sectors (e.g., transport and energy) will increase efficiency, improve resilience, and lower the cost. Although governance capacities varied among cities, management of available information, monitoring and evaluation showed to be reoccurring points for improvement. Cities are also expected to increase implementation capacities using better policy, stricter compliance and preparedness next to promoting community involvement. Consequently, the city transformation process can be more concrete, efficient and inclusive.

## Introduction

At present, over half of the world population lives in cities. The urban population is expected to increase to 66% by 2050, where 6.3 billion people will reside in urban areas. Together with Africa, Asia will have the most rapid urbanisation and, as a result, 52% of the global urban population will be concentrated in Asia in 2050 (UNDESA 2014). By 2030, the world will have an estimated 40% freshwater shortage (UN Water 2018). Many Asian cities depend largely on groundwater. Unsustainable use of groundwater results in land subsidence as observed in Bangkok, Bandung, Jakarta, Ho Chi Minh City (HCMC) and Tokyo (Erkens et al. 2015; WWAP 2015). Cities also generate vast amounts of solid waste. In developing Asian cities, the largest portion of Municipal Solid Waste (MSW) ends in landfills that often lack proper sealing to prevent leaching (Guerrero et al. 2013; Gupta et al. 2015). Without appropriate treatment, solid waste releases hazardous substances that potentially pollute groundwater, surface water and oceans (Jambeck et al. 2015; Zarfl et al. 2011). Discharge of untreated sewage, combined sewer overflows and polluted stormwater runoff, increasingly pollutes Asia’s surface waters. Nutrient emissions in Asia and Africa are projected to double or triple within 40 years, causing serious eutrophication leading to biodiversity loss, and threatening drinking water quality, fisheries, aquaculture and tourism (e.g., Dai et al. 2017; Ligtvoet et al. 2014; OECD Korea Development Institute 2017). Particularly urban areas, including almost all megacities around the world, are vulnerable to both water-related threats and large sources of pollution as well (Ligtvoet et al. 2014). Water pollution affects urban citizen’s health and limits urban economic growth (OECD 2015a; Ligtvoet et al. 2014). Finally, the recovery and reuse of freshwater, energy and materials from wastewater and solid waste are important for realising a circular economy that can address the increasing scarcity of sparse materials, nutrients, freshwater and energy resources (EC 2014; Henckens et al. 2014; Van Leeuwen et al. 2018). Hence, the urban water cycle is crucial for sustainable urban development (Van Leeuwen et al. 2018), which is clearly reflected in the UN Sustainable Development Goals (SDGs) 6 and 11 (UN 2018a).

Governance institutions are faced with a variety of barriers when trying to address above long-term water challenges, which may be summarised as a combination of management fragmentation, technological lock-in, institutional inertia and the challenge of reorienting professional and organisational expertise (Brown and Farrelly 2009; Koop et al. 2017; OECD 2015a; Sydow et al. 2009). These barriers also lead to limited awareness, ill-defined water challenges and a lack of cohesion between short-term targets and long-term goals, as well as inconsistencies between sectors, policies and political agendas (OECD 2015b). Often, water-related measures are taken in an uncoordinated haphazard manner whereby quick fixes are applied, neglecting the co-benefits with other water-related and other sectorial challenges in cities (see Fig. 5 and Table 2 in Koop and Van Leeuwen 2017). Measures should be taken after a thorough diagnosis (baseline assessment) in order to find the most cost-effective and efficient approach. Although an integrated and inclusive approach is often emphasised, there are only few studies that assess urban water management in a consistent and intelligible way (OECD 2015b; 2018). Hence, such an approach is much needed in order to accumulate knowledge and facilitate active engagement of citizens, private stakeholders, professionals and policy makers (Koop and Van Leeuwen 2017).

The City Blueprint^®^ Approach is a diagnosis tool, i.e., the first step in the interactive and strategic planning process of cities to address the challenges of water, waste and climate change (Koop and Van Leeuwen 2015a, b). The approach has been developed as one of the actions of the European Innovation Partnership (EIP) on water, as well as of the Watershare community (EC 2018; Watershare 2017a). It provides an overview of the main components of the urban water cycle by means of 25 performance indicators. At present, 70 cities in more than 35 countries have been analysed based on the approach and a number of publications have been published, presenting the results for the city of Amsterdam (Van Leeuwen and Sjerps 2015), Ahmedabad (Aartsen et al. 2017), Dar es Salaam (Van Leeuwen et al. 2012), Istanbul (Van Leeuwen and Sjerps 2016), Hamburg (Van Leeuwen and Bertram 2013), HCMC (Van Leeuwen et al. 2015), Melbourne (Van Leeuwen 2017), Quito (Scheurs et al. 2018), New York City (Feingold et al. 2018), Seoul (Kim et al. 2018) and Bandung (Rahmasary 2017). Moreover, the European Commission has published the ‘Urban Water Atlas for Europe’ which includes 46 City Blueprints and illustrates the role of water in European cities. The atlas is a novel approach to encourage citizens to understand the relevance of water by combining the work of scientists, artists, politicians and municipal stakeholders with work done by schoolchildren and teachers (Gawlik et al. 2017). Based on this extensive empirical database, key insights have been obtained about the various stages of transformation that cities go through in improving their Integrated Water Resource Management (IWRM) as shown in Table 1. IWRM is defined as a process that promotes the coordinated development and management of water, land and related resources in order to maximise economic and social welfare in an equitable manner without compromising the sustainability of vital ecosystems and the environment (Global Water Partnership 2000).

**Table 1:** Different levels of sustainable IWRM in cities worldwide

BCI (Blue City Index)	Categorisation of IWRM in cities
0–2	*Cities lacking basic water services*
	Access to potable drinking water of sufficient quality and access to sanitation facilities are insufficient. Typically, water pollution is high due to a lack of wastewater treatment (WWT). Solid waste production is relatively low but is only partially collected and, if collected, almost exclusively put in landfills. Water consumption is low, but water system leakages are high due to serious infrastructure investment deficits. Basic water services cannot be expanded or improved due to rapid urbanisation. Improvements are hindered due to governance capacity and funding gaps.
2–4	*Wasteful cities*
	Basic water services are largely met, but flood risk can be high and WWT is poorly covered. Often, only primary and a small portion of secondary WWT is applied, leading to large scale pollution. Water consumption and infrastructure leakages are high due to the lack of environmental awareness and infrastructure maintenance. Solid waste production is high, and waste is almost completely dumped in landfills. Governance is reactive and community involvement is low.
4–6	*Water-efficient cities*
	Cities implementing centralised, well-known, technological solutions to increase water efficiency and to control pollution. Secondary WWT coverage is high and the share of tertiary WWT is rising. Water-efficient technologies are partially applied, infrastructure leakages are substantially reduced, but water consumption is still high. Energy recovery from WWT is relatively high while nutrient recovery is limited. Both solid waste recycling and energy recovery are partially applied. These cities are often vulnerable to climate change, e.g., urban heating and drainage flooding, due to poor adaptation strategies, stormwater separation and limited green surface ratios. Governance and community involvement has improved.
6–8	*Resource efficient and adaptive cities*
	WWT techniques to recover energy and nutrients are often applied. Solid waste recycling and energy recovery are largely covered whereas solid waste production has not yet been reduced. Water efficient techniques are widely applied and water consumption has been reduced. Climate adaptation in urban planning is applied e.g., incorporation of green infrastructures and stormwater separation. Integrative, (de)centralised and long-term planning, community involvement, and sustainability initiatives are established to cope with limited resources and climate change.
8–10	*Water-wise cities*
	There is no city scored within this category so far. These cities apply full resource and energy recovery in their WWT and solid waste treatment, fully integrate water planning and urban planning, have multi-functional and adaptive infrastructures, and local communities promote sustainable integrated decision-making and behaviour. Cities are largely water self-sufficient, attractive, innovative and circular by applying multiple (de)centralised solutions.

The categorisation is based on hierarchal cluster analyses of the City Blueprint and Trends and Pressures analyses in 45 municipalities and regions (Koop and Van Leeuwen 2015b)

To understand how cities can leapfrog through this transformation process, it is pivotal to analyse the main conditions that enable or impede good water governance. In the literature on environmental governance, a plethora of social factors and conditions have been identified that may influence the ability to adapt and respond proactively to the existing and emerging challenges (e.g., Biesbroek et al. 2013; Eisenack et al. 2014). However, despite this rich literature, a comprehensive understanding of the underlying processes that enhance or limit the water governance capacity of cities is largely missing. Governance, transformation and adaptation processes are often not transparent. First, most identified conditions are based on theoretical and conceptual rationales that are not fully validated by empirical data (Biesbroek et al. 2013; Kersberger and Waarden 2004). Second, existing empirical studies are predominantly descriptive or focus on specific case studies that limit their usefulness and learning value beyond the individual context (Measham et al. 2011). Third, concepts, definitions, measurements and methodologies are often inconsistent and not specific (Eisenack et al. 2014).

In this paper, we study the priorities, barriers and enablers of Asian cities to develop their governance capacity necessary for the transformation to address their current and future challenges related to water, waste and climate change (Koop and Van Leeuwen 2017). Section 2 explains the methodologies we apply for our empirical, comparative case study approach. Results are provided in section 3. First, we identify the key challenges in urban water cycle management in 11 Asian cities in order to distinguish management priorities and obtain a thorough understanding of the challenges. Second, we analyse the governance capacity to address these challenges of water, waste and climate change in three Asian cities, Ahmedabad (India), Bandung (Indonesia) and Taipei (Taiwan). This paper focusses primarily on flood risk management as the detailed assessments of other water-related challenges in the city of Ahmedabad, Bandung and Seoul are published elsewhere (Aartsen et al. 2017; Rahmasary 2017; Kim et al. 2018). In section 4, we discuss the value and limitations of our study and embed the results in the literature on urban development and water management in Asia. In section 5, we conclude with identifying the main priorities, barriers and enablers to develop governance capacity to address the challenges in Asian cities.

## Methodology

**Fig. 1:**
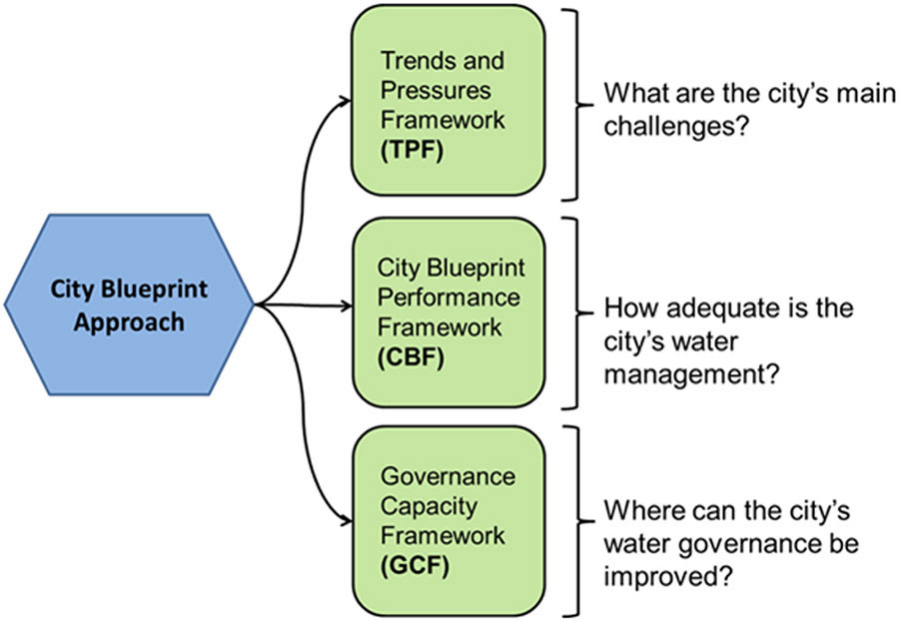
Overview of the City Blueprint Approach comprising three complementary diagnostic assessment frameworks (Koop and Van Leeuwen 2015a, 2015b; Koop et al. 2017)

To identify priorities, barriers and enablers, we applied the City Blueprint^®^ Approach (CBA; Fig. 1). Eleven cities in Asia form part of our empirical study in which we obtain an overview of each city’s main challenges and their performance in addressing these challenges. Detailed information about data sources, calculations and examples are provided in three questionnaires available on the EIP Water website (EC 2018).

### The selection of cities in Asia

**Fig. 2:**
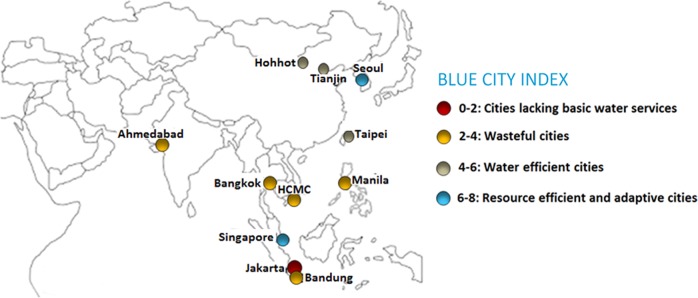
Map with eleven Asian cities included in the City Blueprint study. The cities’ categorisation is in accordance with Table 1

In order to study Asia’s urban water management, 11 cities were selected that represent the rapid transformation of Asian cities, including a diversity of cities at different stages of this transformation (Table 1). The selection of cities for this study was not random. It was made based on existing collaborative research networks of the University of Utrecht and KWR Watercycle Research Institute, e.g., Watershare^®^ (Watershare 2017a, b), where we tried to include a representative selection of cities in a variety of Asian countries. All the selected cities (Tables 2 and 3; Fig. 2) are rapidly expanding. Most of them are located along the coast (e.g., Bangkok and Jakarta), islands (e.g., Singapore and Taipei) and some are inland (e.g., Ahmedabad and Hohhot). The differences in geographical setting and level of Gross Domestic Product (GDP) reflect the diversity in the continent and allows for the identification of priorities, barriers and enablers to develop the capacity to transform water management in Asian cities.

**Table 2:** Key characteristics of 11 Asian cities assessed by the City Blueprint Approach

City	Population size^a^	Average urbanisation rate 2000–2016 (% Year^−1^)^b^	GDP per capita (current US$)^c^
Ahmedabad	3,719,710	+2.37	1939.6
Bandung	1,699,719	+2.27	3846.9
Bangkok	5,104,476	+1.73	6593.8
Hohhot	774,477	+2.42	8827.0
Ho Chi Minh City	3,467,331	+2.98	2343.1
Jakarta	9,607,787	+2.27	3846.9
Taipei	7,871,900	+0.80	8827.0
Tianjin	11,090,314	+2.42	8827.0
Manila	1,600,000	+1.99	2989.0
Seoul	10,349,312	+0.30	29,742.8
Singapore	3,547,809	+1.39	57,714.3

^a^World Population Review http://worldpopulationreview.com/. Note that urban agglomerates can be larger

^b^Central Intelligence Agency https://www.cia.gov/library/publications/the-world-factbook/fields/2212.html. Country average urbanisation rate

^c^World Bank https://data.worldbank.org/indicator/NY.GDP.PCAP.CD

### Trends and Pressures Framework (TPF)

The TPF consists of 12 descriptive indicators to summarise the exogenous social, environmental and financial conditions within which water managers have to operate (Table 3). Each indicator is scaled from 0 to 4 points, where a higher score represents a higher urban pressure or concern (Koop and Van Leeuwen 2015a). Most scores of the indicators are based on national quantitative data from, for example, the World Bank, World Health Organisation and the Food and Agricultural Organisation. Most indicator scores are determined using the ranking of the city amongst all available country scores and the average of all indicators is called the Trends and Pressures Index (TPI). It provides an indication of the urban pressures with respect to global trends. Detailed information on the scoring methods is provided by Koop and Van Leeuwen (2015a, b) and the EIP Water website (EC 2018).

**Table 3:**
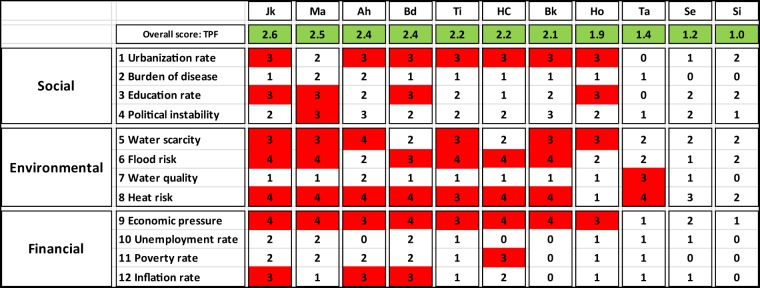
Comparison of TPF indicator scores analysing the social, environmental and financial pressures in Jakarta (Jk), Manila (Ma), Ahmedabad (Ah), Bandung (Bd), Tianjin (Ti), HCMC (HC), Bangkok (Bk), Hohhot (Ho), Taipei (Ta), Seoul (Se), Singapore (Si). Concerns (score = 3) and high concerns (score = 4) are highlighted in red

### The City Blueprint Framework (CBF)

The CBF consists of 25 indicators divided into seven categories: (1) water quality, (2) solid waste treatment, (3) basic water services, (4) wastewater treatment, (5) infrastructure, (6) climate robustness, and (7) governance. Each indicator is scored from 0 (much room for improvement) to 10 (best practice), where their geometric mean is called the Blue City Index (BCI). The CBF uses scientific articles, websites and official reports at the city level as its source for calculating or assigning a score for each indicator. Detailed information about data sources, calculation methods, scaling methods and limitations of the CBF are provided by Koop and Van Leeuwen (2015a) and the EIP Water website (EC 2018).

### The Governance Capacity Framework (GCF)

The GCF analyses the governance capacity of a city to address a specific common water challenge (Koop et al. 2017; Watershare 2017b). The GCF provides information on how well different organisations can work together and to what extent capacities are institutionalised. In this way, governance capacity is understood as the result of human skills and expertise. In its more recent extension, the GCF has been applied in 15 cities where the publications of Quito (Scheurs et al. 2018), New York City (Feingold et al. 2018), Sabadell (Steflova et al. 2018), Ahmedabad (Aartsen et al. 2017), Seoul (Kim et al. 2018), Bandung (Rahmasary 2017), Cape Town (Madonsela et al. 2019) and Amsterdam, Rotterdam, Leicester and Milton Keynes (Koop et al. 2018) are available online. The GCF consists of three dimensions and nine conditions (Table 4). Each condition has three indicators that are scored using a Likert scale ranging from very encouraging (++) to very limiting (− −) the overall governance capacity to address a water challenge. A detailed description of the Likert scale for each of the 27 indicators is available (EC 2018). Water governance is assessed with respect to five challenges: (1) flood risk, (2) water scarcity, (3) solid waste treatment, (4) wastewater treatment and (5) urban heat islands (UHI). The indicator scoring was done through a triangular approach to validate findings by different sources in three consecutive steps:

1. Desk study of policy documents, scientific literature and grey literature to provide a preliminary score for each of the 27 indicators based on substantiated argumentation and references to the studied material.
2. Interviews with relevant local stakeholders. Stakeholders in each city were analysed and categorised to ensure the representativeness of interviewees and the diversity of water managers. This paper categorised the stakeholders based on their importance/influence, functionality or power/interest. Multiple interviewees from identified stakeholders were selected for in-depth interviews to collect information, score the indicators and ask follow-up questions for clarification or a better overall understanding. To minimise the risk of bias and assure diversity amongst interviewees, they were selected according to their roles, expertise and responsibilities. Based on the interviews, the preliminary indicator scores were updated.
3. Feedback from interviewees. Interviewees were asked to provide constructive feedback on the updated indicator scores. After including this feedback, the final scores were determined.

**Table 4:**
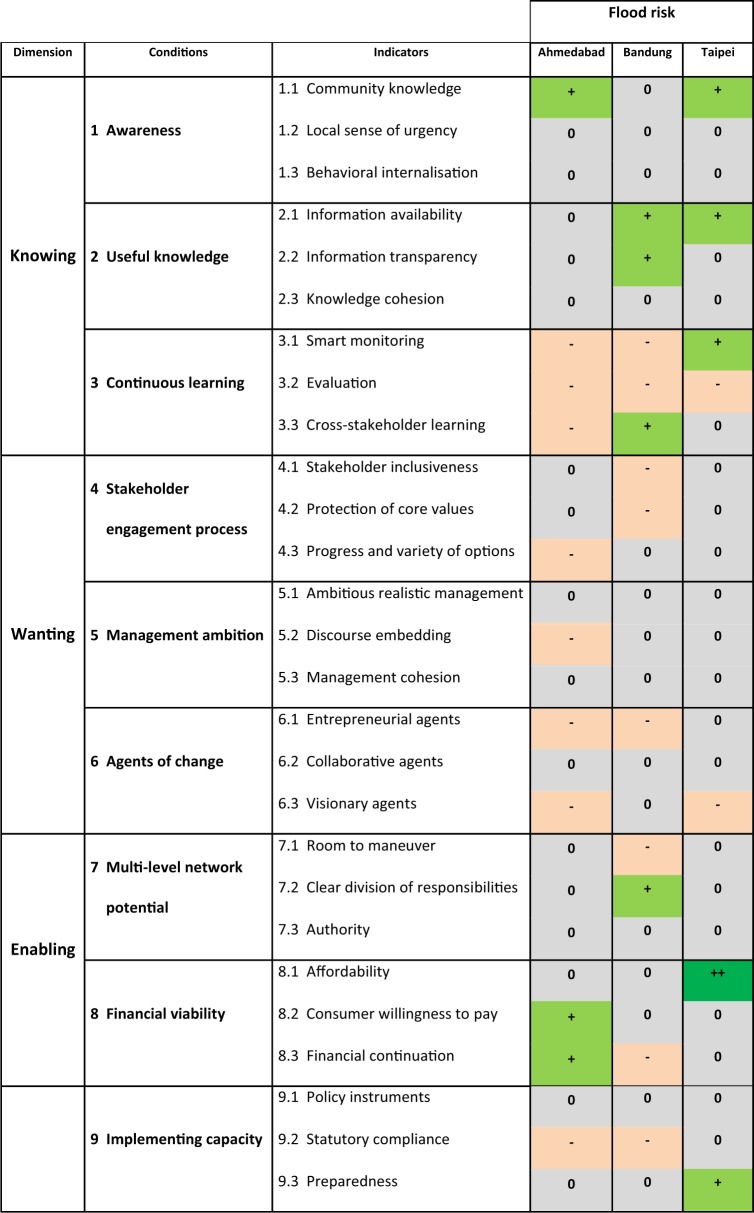
Governance Capacity profile of three Asian cities with respect to the integrated challenge of flood risk (Aartsen et al. 2017; Rahmasary 2017; Robert 2017)

## Results

### Case Selection: Comparison of Social, Environmental and Financial Settings in Asian Cities

Asia’s coasts urbanise at a staggering rate. In fact, 11 of the world’s 17 largest cities are coastal Asian cities, such as Bangkok, Jakarta or Manila (Tibbetts 2002). Economic growth is lifting hundreds of millions of people out of poverty and a rapidly growing urban middle class accounts for about two billion people at present. However, Asian cities also include the world’s largest urban slum populations and the largest population living below the poverty line in areas vulnerable to environmental risks, such as floods, landslides and the effects of pollutions (UN-HABITAT and ESCAP 2015). The variety in social, environmental and financial contexts of these cities is reflected in the pressures that these cities experience (Table 3).

In general, many cities in Asia, especially those with low average GDP and high inflation, face social pressure, particularly coming from high urbanisation rates, environmental pressure from water scarcity, pollution, flooding and heat risk, as well as financial pressure. During several decades, the expansion of the global market in Asia transformed its large cities into population magnets with relatively high economic and urban growth (Douglass 2010; Firman 2009). Coastal cities like HCMC, Jakarta and Bangkok are recurrently used as case studies of sinking cities with an average land subsidence rate of 20–100 mm/year (Erkens et al. 2015). Uncontrolled groundwater abstraction in Bandung and Taipei aggravates their land subsidence to 80 mm/year (Abidin et al. 2013; Hwang et al. 2016). The two least pressured cities, Seoul and Singapore, face freshwater scarcity. Nevertheless, both cities secure their drinking water supply using advanced technology. Seoul’s water works authority purifies 3.19 million m^3^ drinking water from the Han River, while Singapore is known for its four national taps of imported water, local rainwater harvesting, NEWater (reclaimed water) and desalinated water (SMG 2014; PUB 2017). Many cities in Asia face heat risks. With the most recent heat-wave temperatures of 48 °C in 2016, Ahmedabad’s inhabitants faced an ever-growing heat risk (Aartsen et al. 2017). Overall, cities with high social pressures, such as Jakarta and Manila, tend to have high financial pressures. In fact, the city’s overall social, environmental and financial pressures reflected in the TPI has been found to correlate negatively with the IWRM performance reflected in the BCI (Koop and Van Leeuwen 2015b).

### Overview of IWRM Practices in Asian Cities

Figure 2 shows the results of the City Blueprint analyses in the selected cities according to categorisation shown in Table 1. The BCI scores span a large range of 2.0 to 8.1. Jakarta, HCMC, Bandung, Bangkok, Manila and Ahmedabad are grouped among cities with low BCIs. According to Table 1, these six cities can be categorised as *wasteful cities* (BCI 2–4), where basic water services are mostly covered but flood risk exists and waste management is poor. Taipei, Tianjin and Hohhot are categorised as *water efficient cities* (BCI 4–6) where infrastructure and technologies in addressing basic water services, solid waste and wastewater treatment are more developed. Finally, Seoul and Singapore have implemented measures to be self-sufficient and circular. They categorise as *resource efficient and adaptive cities* (BCI 6–8).

**Fig. 3:**
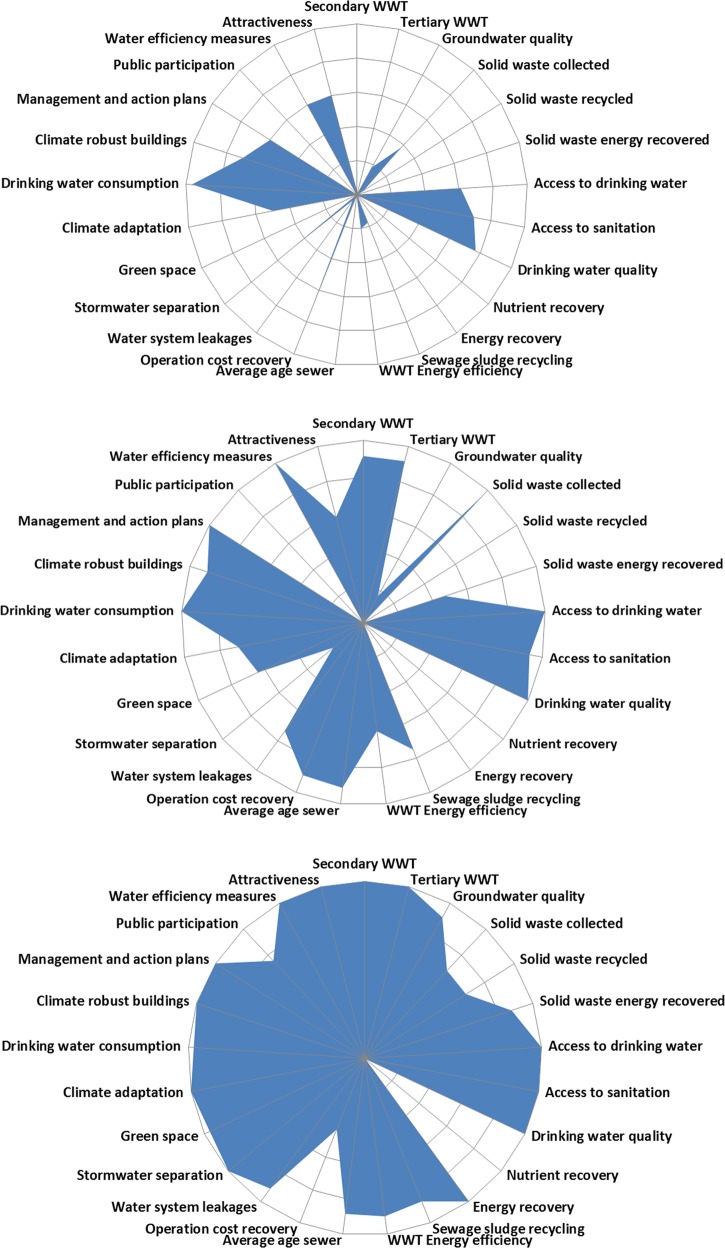
Spider diagrams based on 25 performance indicators for Jakarta (top), Tianjin (centre) and Singapore (bottom). The BCI, the geometric means of the 25 indicators, are 2.0, 4.9 and 8.1, respectively

Figure 3 shows the City Blueprint’s spider diagrams of Jakarta (BCI 2.0), Tianjin (BCI 4.9) and Singapore (BCI 8.1). Jakarta’s lowest scores are mostly in the category of water quality, solid waste treatment and wastewater treatment. Similarly, HCMC (BCI 2.4), Bandung (BCI 2.6), Bangkok (BCI 2.6), Manila (BCI 2.6) and Ahmedabad (BCI 3.0) have good basic water services, low drinking water consumption, low coverage of secondary WWT systems and combined sewers. These cities have difficulties in meeting their basic water services for marginalised communities. In particular HCMC has low access to sanitation. Moreover, in these cities, wastewater is typically insufficiently collected and treated. Drinking water consumption is low and water leakages are high. Jakarta has the largest water system leakage rate of 48%. Ahmedabad and Manila score higher in solid waste treatment while HCMC has a relatively new sewerage network. Tianjin, Taipei (BCI 3.9) and Hohhot (BCI 5.0) have fully covered basic water services, good solid waste collection systems and high coverage of WWT, but no nutrient and energy recovery yet. Moreover, Taipei has a high drinking water consumption of 342 m^3^/person/year. Meanwhile, Seoul (BCI 7.3) and Singapore (8.1) have excellent WWT systems with full coverage and efficiency, high percentage of energy recovery from solid waste incineration and implement adaptation plans to improve the city’s climate robustness.

### Governance Capacity to Address Flood Risk

In order to understand the main barriers and enablers that Asian cities experience in their transformation trajectory, we have analysed the water governance capacity of three transforming cities: Ahmedabad, Bandung and Taipei. In this section, we only provide a short overview and mainly limit ourselves to the governance capacity to deal with the risk of flooding. Table 4 displays a graphic view of the governance capacity profile in these three cities.

#### Ahmedabad

In Ahmedabad, visionary agents (ind. 6.3) within the government use their authority (ind. 7.3) to set ambitious goals (ind. 5.1). However, insufficient statutory compliance (ind. 9.2) and poor use of policy instruments (ind. 9.1) limit the implementation of these goals. In particular, insufficient monitoring (ind. 3.1) and policy evaluation (ind. 3.2) hinder learning for better implementation practices. Awareness and financial viability (conditions 1 and 8) are found to encourage governance capacity. On the other hand, continuous learning and implementing capacity (conditions 3 and 9) reduce the governance capacity needed to address flood risks. Interestingly, Ahmedabad’s Governance capacity to address heat risks was found to be rather encouraging (Aartsen et al. 2017). This can be seen from the success case of Ahmedabad’s Heat Adaptation Plan. During the 2010 heat wave, an excess of 1,344 additional deaths in Ahmedabad were reported, i.e., an increase of 43.1% compared with the previous years (Shah et al. 2014). It sets an example for the city to improve its governance capacity to address other challenges of water, waste and climate change.

#### Bandung

Flooding in Bandung has increased in frequency where during the worst seasonal flood, it can be stagnant for 2–4 weeks and causes temporary evacuation. Flooding is the main reason for increased traffic congestion contributing to the city’s air pollution. Inappropriate solid waste collection and treatment causes clogs in drainage channels, which exacerbate flooding events even further (Rahmasary 2017). Bandung can improve its smart monitoring and evaluation process (condition 3). The city’s statutory compliance (ind. 9.2) to existing legislation, policies and agreements can be considered a priority for improvement. In addition, sharing knowledge and practice can be improved by endorsing collaboration among engaged stakeholders and multi-level networking (condition 4). Agents of change (condition 6) play a critical role in raising public awareness and realising behavioural change (condition 1) especially in solid waste and wastewater treatment challenges (Rahmasary 2017). Additional support from the regional and national government can be used more effectively to address the city’s strongly interrelated water challenges.

#### Taipei

In Taipei, public awareness of flood risk (ind. 2.1) and smart monitoring (ind. 3.1) is relatively high. Equitable financial support for climate protection (condition 8) is largely provided. Taipei has learned from past disasters. Monitoring systems are further developed and improved, and companies and citizens comply with taxes to further reduce flood risks. However, these measures are merely focussed on short-term flood risks. To further improve the long-term governance capacity for Taipei, the city needs to elaborate detailed long-term urban planning (ind. 5.1) and support visionary agents (6.3). The agents of change, such as the Ministry of Science and Technology, the Taiwan Youth Climate Coalition, private companies and universities, should explore opportunities that go beyond technical solutions and the government and the private sector should provide financial resources to support these entrepreneurial activities. This broader approach may contribute and further improve the city’s preparedness. In particular, capacity development may need to focus on:

1. Information transparency and comprehensiveness for everyone (ind. 2.2 and 2.3);
2. Awareness raising by applying bottom-up approaches (ind. 1.2);
3. Integration between different levels and different stakeholders (condition 7);
4. Improving the evaluation process to include long-term risks and climate change (condition 3)

## Discussion

### Method Validity and Limitations

City-level data are scarce globally. This limited availability also has its effects on the accuracy of the TPF and CBF indicators because, for some of the CBF indicators, national-level data are used for the calculations (Koop and Van Leeuwen 2015b). Because the method aims to enhance city-to-city learning worldwide, the framework also includes only those indicators for which data can be obtained relatively easily. Hence, additional city-specific features can be added and included in the City Blueprint reports. For the GCF, it can be argued that a lack of knowledge or experience of an interviewee will affect the assessment validity. Therefore, the desk study is of critical importance to check statements of interviewees. Moreover, the stakeholder analysis is also crucial to ensure inclusion of all key stakeholders and select adequate interviewees. To make sure this study is reproducible and reliable, GCF analyses in each city are supported by a justification report with detailed substantiation for each indicator score based on desk studies, interviews and feedback from the interviewees. The interviewees are anonymous to avoid socially desired answers. Finally, the TPF, CBF and GCF provide only an overview (snapshot) of the current conditions. A repetition of the analyses over time using these assessments is advised to monitor the indicators and to capture their trends over time.

**Fig. 4:**
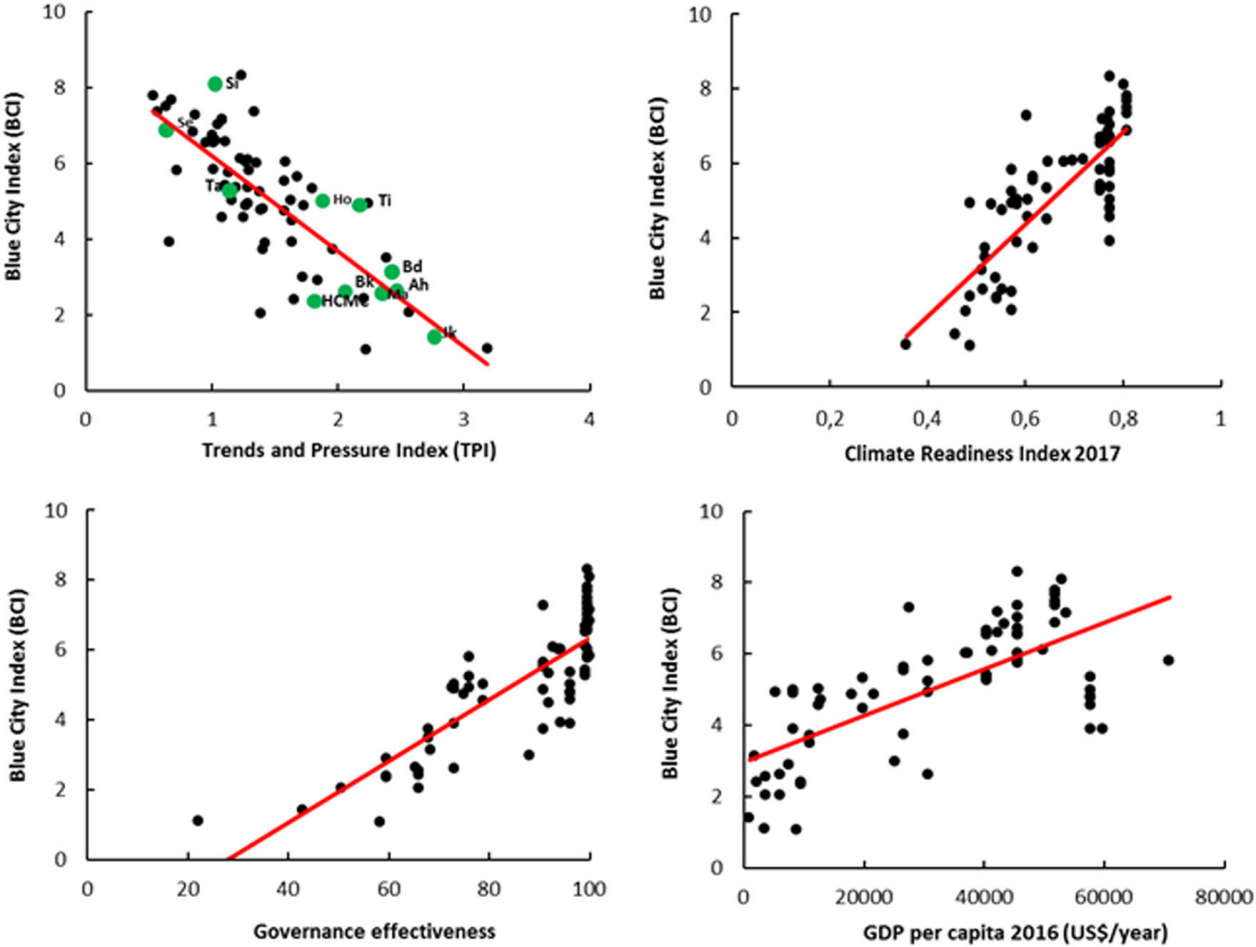
Correlation of the BCI with the TPI, climate readiness index (ND-GAIN 2018), government effectiveness (World Bank 2018) and GDP per capita (IMF 2017). The correlation coefficients are, respectively, −0.79, 0.80, 0.80 and 0.70

In general, the CBA provides a good starting point to improve IWRM for cities and simultaneously allows for standardisation and reproducibility of the results. The overall scores of the TPF and CBF assessments in cities provide a unique frame to analyse global patterns of urban water management, such as the categorisation of IWRM (Table 1). Moreover, correlations with other indicators can be explored in order to identify patterns and possible causalities that need to be validated through more advanced methods. For example, Fig. 4 provides interesting correlations of the overall BCI score in 70 cities with TPI (r = −0.79), the climate readiness index (r = 0.80), governance effectiveness index (r = 0.80) and GDP per capita (r = 0.70), respectively.

Cities with high pressures (high TPIs) usually have low BCIs, most probably because they face more constraints and challenges in attaining good water management, which is in line with earlier observations (Koop and Van Leeuwen 2015a). The climate readiness index measures the ability to absorb and mobilise financial support for climate change adaptation. Its high correlation with the BCI means that cities with good IWRM performance are also climate-ready (Koop and Van Leeuwen 2015b). Governance capacities may also be low in developing cities, due to their high social and financial pressures. Unfortunately, the current number of cities (*n* = 15) analysed by the GCF is insufficient to calculate valid correlation coefficients. The BCI correlates well with the GDP per capita of a country (r = 0.70), which makes sense as there are more financial resources to invest in urban water management. However, the BCI correlates better with The World Bank’s indicator *governance effectiveness* of countries. The differences in BCI between cities within a country can be rather substantial, meaning that these correlation coefficients have limited empirical value, but they still do provide one of the most accurate indications available for developments in urban IWRM.

### The Transformation Trajectory of Asian’s Urban Water Management

Most cities—HCMC (BCI 2.4), Bandung (BCI 2.6), Bangkok (BCI 2.6), Manila (BCI 2.6) and Ahmedabad (BCI 3.0)— are classified as ‘*wasteful*' according to the IWRM categorisation shown in Table 1. Indeed, these cities often face substantial flood risks, poor WWT and solid waste treatment that all lead to large scale pollution. Drinking water leakage is relatively high due to insufficient investments in the refurbishment or replacement of existing infrastructure. The classification as shown in Table 1 is largely based on wasteful cities in Eastern Europe. The six Asian cities show similar features. However, there is also a large difference: cities in Eastern Europe have stable or decreasing populations, whereas Asian cities are rapidly growing. In contrast to cities in Eastern Europe, access to basic water services, such as access to improved sanitation and drinking water, is not ensured in the informal settlements that emerge due to rapid urban expansion. In addition, drinking water consumption in many of these Asian cities was low due to limited piped water supply, whereas this does not apply to Eastern European cities. It is therefore necessary to reassess the categorisation proposed by Koop and Van Leeuwen (2015b; Table 1) that was largely developed with data biased towards Europe. The cities of Tianjin, Taipei (BCI 3.9) and Hohhot (BCI 5.0) comply well with the category ‘*water efficient city*’ as they apply well-known, centralised technological solutions to increase water efficiency and to control pollution. The cities of Seoul (7.3) and Singapore (8.1) apply advanced techniques to reduce climate vulnerability, recover resources and conserve water. In particular, Singapore has already implemented effective holistic urban planning and has almost transformed itself to be a ‘*water wise city*’.

The challenge of flood risk intertwines strongly with other issues, such as solid waste management, wastewater treatment and water scarcity. Uncollected solid waste clogs drainage systems leading to flooding and water pollution. Poor wastewater treatment also leads to water pollution, threatening human health, fish populations and clean water availability as such. IWRM is of critical importance in these rapidly expanding Asian cities and a proper understanding of the actual risks and effectiveness of measures is key. Accordingly, the governance capacity of cities to transform their water management can be considered as a key area of research (e.g., Koop and Van Leeuwen 2017; OECD 2015a, 2015b). In the comparative analysis of the capacity to govern flood risk in the city of Ahmedabad, Bandung and Taipei, some priorities have been identified that may also apply to other cities in Asia. In particular, the evaluation process of existing policy and practices is a key point to improve as it is a precondition for learning and improved implementing capacity. All three cities face issues of statutory compliance which can be understood in light of the many social, environmental and financial pressures they face, especially with rapid urban expansion. The GCF findings indicate that national governments in Asian cities largely use top–down approaches in decision-making processes of the water management sector. This practice can be improved by combining top–down and bottom-up approaches. It creates a consultative state using a two-way flow of advice to combine expert and local personal experience, also to ensure that community needs are identified and addressed (Kobayashi and Porter 2012). Nowadays, more cities are exploring a combined approach by providing offline and online platforms that allow citizens to be involved in urban planning activities. This transition to a cross-stakeholder water governance approach helps local governments to receive broader support and acceptance in their decision-making process (Perreault 2014).

### Municipal Solid Waste and Wastewater Challenges

Major improvements are needed in developing cities with respect to MSW management and WWT. A change in public behaviour is required, as many citizens often dispose their solid waste and wastewater directly on streets, in poorly maintained landfills and rivers. Industrial activities are also known as a major source of water pollution for many urban rivers in China, India and Indonesia (Fulazzaky 2014; Herricks and Suen 2003; Maheshwari 2016). Cities with sufficient financial resources often do collect, separate and process their MSW. South Korea changed their landfilling practices and promoted recycling and incineration. In 2010, 60.5% of their MSW was recycled and 21.6% was incinerated (Min and Rhee 2014). Singapore also cuts their landfilling practice due to limited space. At present, 21% of Singapore’s MSW is recycled and 69% is incinerated (NEA 2016). Still, the largest percentage of MSW in developing Asian cities ends up in landfills. However, most landfills are not properly designed as a sanitary landfill (e.g., lacking of durable plastic or clay-lined layers to prevent leaching) (Guerrero et al. 2013; Gupta et al. 2015). The practice of solid waste separation for composting and recycling has risen in Asian cities with average MSW composition of 40–60% organic waste. Cities in India treat their organic MSW by composting, vermi-composting (using worms) and anaerobic digestion (producing methane and manure; Gupta et al. 2015). In Indonesia, waste banks increase the public willingness to dispose of their MSW separately, using financial incentives. These banks pay for people’s valuable waste (i.e., plastic, metal, paper) to be recycled (Dhokhikah et al. 2015). South Korea uses regulations on the restricted use of disposable products, i.e., a volume-based waste fee system (VBWFS) and food waste recycling. The introduction of VBWFS led to 17.8% reduction in MSW generation and a 26% increase in recyclable wastes in the first year of 1995, and overall reduction of MSW generation by 14% per year during the period of 1994 to 2004 (Kim and Kim 2012). The government also issued Extended Producer Responsibility that invokes producers to reduce their products’ packaging (Min and Rhee 2014).

Wastewater disposal and treatment systems in developing cities are underdeveloped. They often include aerated lagoons, septic tanks and latrines (Varis 2006). The number of wastewater treatment plants is increasing globally, but this development is unable to keep up with the rapid population growth and urbanisation observed in many cities in Asia. This off-site centralised treatment is preferable to improve public health and to reduce environmental contamination. However, local regulations, land availability and a lack of financial resources to build sewer infrastructure are reoccurring constraining factors (Kerstens et al. 2015). The slow progress in developing centralised treatment plants results in more attention for the ‘community as users’ principal, e.g., community-based sanitation (CBS) and decentralised wastewater treatment system programmes (DEWATS; Prihandrijanti and Firdayati 2011; Sofyan et al. 2016). CBS and DEWATS programmes are demand-driven approaches with high community involvement. They require less extensive infrastructural investments. Still, continuous supervision and improvements are needed to ensure the quality of the treatment units since they are known to have comparably low removal efficiencies (Kerstens et al. 2015). It is also important to provide local communities with regular assistance in using and maintenancing these services.

### The Role of Community Movements in Alleviating Basic Water Services in Slums

**Fig. 5:**
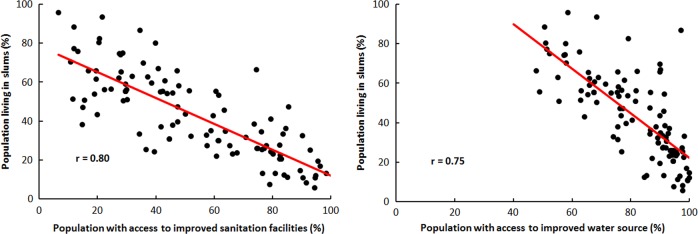
Population living in slums (%) for each country in relation to improved sanitation facilities (%; left) and access to improved water sources (%; right). Data from the World Bank (2014, 2015a, 2015b)

Citizens living in informal settlements (slums) constitute a significant percentage of the urban population. A recent estimate states that 32.7% of the world’s population in developing regions is living in slums (UN-HABITAT 2011). There were more slum dwellers in 2012 than in 2000, a trend that will likely continue in the future (WWAP 2017). Slum dwellers frequently rely on unsewered communal toilets, use open spaces or dispose of faeces in polythene bags. The inequality in sanitary services is significant. For instance, in India 56% of the population in the top 20% (household-income groups) has access to piped water, compared with 6% of the bottom 20% (World Bank 2017). Rapid urban expansion aggravates these challenges and the people are also disproportionately affected by the impacts of climate hazards (Jamil 2013; UN-HABITAT 2013; Varis 2006; WWAP 2017). The proportion of the urban population living in slums in China, India, Indonesia, Philippines and Thailand is, respectively, 25, 24, 22, 38 and 25% (UN 2018b). Furthermore, groundwater dependency is high in Asia, especially throughout South Asia and China. Approximately one-third of Asia’s population (some 1 billion to 1.2 billion people) is reliant on groundwater (Hirji et al. 2017; WWAP 2018). The provision of basic water services (drinking water and sanitation and wastewater treatment) in informal settlements faces an even greater challenge (WWAP 2017, 2018). Figure 5 shows the relation between population living in slums with access to improved sanitation and improved water sources.

In general, the highest percentage of populations living in slums are in cities that are categorised as cities *‘lacking basic water services’*, followed by cities belonging to the category *‘wasteful cities’* (Table 1). These cities include Belem (Brazil), Kilamba Kiaxi (Angola), Dar es Salaam (Tanzania), Quito (Ecuador), Jakarta (Indonesia), (Bulgaria), HCMC (Vietnam), Bandung (Indonesia), Bangkok (Thailand), Manila (the Philippines), Varna (Bulgaria) and Ahmedabad (India). These results emphasise the need of alleviating the slum’s provision to attain better water and wastewater services. These are also major global goals embedded in the UN SDGs (UN 2018a).

Special budgets from governments are often available for slum alleviation programmes, next to available local resources, since slum populations in developing countries often comprise the largest part of the informal sector in urban economic development (Jamil 2013). However, despite available programmes such as relocation, affordable flats, CBS and other incentives, the local community is often reluctant to change their current living situation (Jamil 2013). To address this situation, local authorities should understand the characteristics of local slums (UN-HABITAT 2011). For example, engaging slum dwellers early on in local decision-making increases their ‘*sense of belonging’* and willingness to cooperate. A continuous commitment is required to realise proper implementation of these programmes (Mol 2009; Suharko 2015). These local community movements generally aim for smaller and more accurate targets. Importantly, they represent the interests and core values of the marginalised communities and play the role of a mediator for the government (Suharko 2015). For example, Bandung’s civil society organisations are very collaborative by expanding public engagement using social media platforms to raise awareness in slums (Rahmasary 2017).

## Conclusion

This paper focuses on the priorities, barriers and enablers in Asian cities to develop the governance capacity necessary to address their challenges related to water, waste and climate change. The results show a great variety in performances and capacities reflected by the high variation of the BCI scores of Asian cities varying from 2.0 to 8.1. This large variation emphasises their learning and transformation potential, provided that cities exchange knowledge, experiences and best practices. Cities with more social, environmental and financial pressures were found to have lower water management performances. On the other hand, the cities of Seoul (BCI 7.3) and Singapore (BCI 8.1) are world leading examples of water conservation, circular urban water management and climate robustness. The main priorities observed in Asian cities were water scarcity, water leakage, flood and heat risk, wastewater treatment and solid waste collection and recycling (Table 3 and Figs. 2 and 3). The main barriers observed were adequate education and good governance (Tables 3 and 4). Our study shows that there are several options for improvement:

- The main enablers reside in adequate education and good governance: ‘before fixing the urban water pipes, fix the institutions.' (OECD 2016). In Asia, this specifically includes the need for proper monitoring, cross-stakeholder learning, implementation and enforcement as well as sufficient room for new initiatives (Table 4).
- This study in Asian cities reconfirms our earlier observations that water challenges form a cross-cutting issue that require a holistic rather than a sectorial approach in order to create co-benefits and win-wins (Koop and Van Leeuwen 2017). Defragmentation of institutions may play a key role again.
- Based on the governance capacity analysis of flood risk management in the cities of Ahmedabad, Bandung and Taipei, we conclude that improvements in both the statutory compliance and policy evaluation processes can be considered as key priorities as well.
- Proper IWRM is often hindered by low performance in solid waste collection and recycling leading to clogging of sewers, and subsequently to high risks of both flooding (after heavy rain events) and water scarcity (as water reuse requires proper collection and treatment of wastewater). Thus, proper IWRM should encompass adequate solid waste management.
- IWRM in slum areas is of particular importance for Asia’s rapidly expanding cities. Here, citizen engagement is an important precondition for their sustainability and resilience. Improvements can only be realised through inclusive local decision-making and long-term commitment. In particular, access to basic water services, solid waste management and various forms of centralised and decentralised wastewater treatment can be considered as focal areas.
